# High Prevalence of Late-Onset Fabry Cardiomyopathy in a Cohort of 499 Non-Selective Patients with Left Ventricular Hypertrophy: The Asian Fabry Cardiomyopathy High-Risk Screening Study (ASIAN-FAME)

**DOI:** 10.3390/jcm10102160

**Published:** 2021-05-17

**Authors:** Yiting Fan, Tsz-Ngai Chan, Josie T. Y. Chow, Kevin K. H. Kam, Wai-Kin Chi, Joseph Y. S. Chan, Erik Fung, Mabel M. P. Tong, Jeffery K. T. Wong, Paul C. L. Choi, David K. H. Chan, Bun Sheng, Alex Pui-Wai Lee

**Affiliations:** 1 Cardiology Department, Shanghai Chest Hospital, Shanghai Jiaotong University, Shanghai 200030, China; myricefyt@126.com; 2 Laboratory of Cardiac Imaging and 3D Printing, Li Ka Shing Institute of Health Science, The Chinese University of Hong Kong, Hong Kong, China; jimchan202005@gmail.com (T.-N.C.); josiechow@cuhk.edu.hk (J.T.Y.C.); 3 Division of Cardiology, Department of Medicine and Therapeutics, The Chinese University of Hong Kong, Hong Kong, China; kkh381@ha.org.hk (K.K.H.K.); cwk743@ha.org.hk (W.-K.C.); cys644@ha.org.hk (J.Y.S.C.); e.fung@cuhk.edu.hk (E.F.); 4 Department of Radiology, Alice Ho Miu Ling Nethersole Hospital, Hong Kong, China; tmp678@ha.org.hk; 5 Department of Imaging and Interventional Radiology, The Chinese University of Hong Kong, Hong Kong, China; wongkatakjeffrey@hotmail.com; 6 Department of Anatomical and Cellular Pathology, The Chinese University of Hong Kong, Hong Kong, China; paulchoi@cuhk.edu.hk; 7 Clinical Genetic Service, Department of Health, Hong Kong, China; DavidChan68@hotmail.com; 8 Princess Margaret Hospital, Hong Kong, China; shengb@ha.org.hk

**Keywords:** fabry disease, left ventricular hypertrophy, IVS4 + 919G > A mutation, dried blood spot test

## Abstract

Left ventricular hypertrophy (LVH) caused by cardiac variant Fabry disease (FD) is typically late-onset and may mimic LVH caused by abnormal loading conditions. We aimed to determine the prevalence of FD in a non-selective patient population of everyday practice presenting with LVH, including those with hypertension and valve disease. We measured plasma alpha-galactosidase A activity using dried blood spot tests in 499 (age = 66 ± 13 years; 336 men) Hong Kong Chinese patients with LVH defined as maximal LV septal/posterior wall thickness ≥13 mm on echocardiography. Patients with low enzyme activity underwent mutation analysis of the GLA gene. Eight (age = 53−74 years; all men) unrelated patients (1.6%) had low plasma alpha-galactosidase A activity (0.57 ± 0.27 μmol/L wb/hr) and all were confirmed to have the GLA IVS4 + 919G > A mutation. FD patients presented with heart failure (*n* = 5), heart block (*n* = 2), ventricular tachycardia (*n* = 1), chest pain (*n* = 3), and/or murmur (*n* = 1). Uncontrolled hypertension (*n* = 4) and/or severe mitral/aortic valve pathology (*n* = 2) were frequent. Ethnic subgroups included Teochew (*n* = 5), Canton (*n* = 2), and Wenzhou (*n* = 1). Endomyocardial biopsy (*n* = 6) revealed hypertrophic myocytes with vacuolization and dense lamellar bodies. Late-onset IVS4 + 919G > A FD is prevalent among Chinese LVH patients, and should be considered as a cause of LVH in adult patients even when hypertension and/or valve pathology are present.

## 1. Introduction

Fabry disease (FD) is an X-linked, inborn error of glycosphingolipid metabolism resulting from mutations in the *GLA* gene in the X chromosomal region Xq22.1 that leads to deficient activity of the lysosomal enzyme, alpha-galactosidase A [1,2,3]. Deficiency in alpha-galactosidase A leads to accumulation of globotriaosylceramide in the vascular endothelium and other cell types of various organs including the skin, heart, kidney, eyes, nerves, and brain [2]. The classic form of FD typically affects multiple organs manifesting clinically with acroparesthesias, angiokeratomas, hypohidrosis, and corneal dystrophy during childhood or adolescence [4,5]. The later-onset Fabry phenotype, on the other hand, does not usually display the classical features of FD, and is typically dominated by a particular organ system, most commonly the heart [4]. As enzyme replacement therapy (ERT) is associated with better outcomes when given in an earlier disease stage, timely diagnosis and early ERT initiation has become increasingly important for FD patients and their family members.

The worldwide incidence of FD is estimated to range from 1:40,000 to 1:117,000, but is likely an underestimation as a result of delayed or missed diagnoses [1]. There is reportedly an exceptionally high incidence of an FD-associated intervening sequence mutation, the IVS4 + 919G > A mutation, among East Asian populations that is not common in Western populations. This intervening sequence mutation that results in aberrant alternative splicing [6] is prevalent in Japan [7] and Taiwan [8] and perhaps originated in ancient China [9]. Patients with IVS4 + 919G > A cardiac variant FD typically present with left ventricular hypertrophy (LVH) during the fifth to the eighth decade of life, a time in life when acquired causes of LVH-like systemic hypertension and valvular heart disease is also frequent. Previous studies screening for FD among LVH patients predominantly targeted their screening efforts at patient populations with idiopathic LVH, excluding patients with abnormal loading conditions potentially causing LVH such as severe aortic stenosis and uncontrolled hypertension [10,11,12]. Given the high prevalence of the hotspot IVS4 + 919G > A mutation among Asian populations and that the cardiac variant of FD cannot be clinically differentiated from, and thus may mimic, other more frequently acquired causes of LVH in adulthood, the present Fabry Cardiomyopathy High-Risk Screening Study (ASIAN-FAME) is conducted to determine the true prevalence of FD in a non-selective Asian Chinese population of everyday clinical practice presenting with LVH, without exclusion of patients with abnormal loading conditions including systemic hypertension and valvular heart disease.

## 2. Materials and Methods

### 2.1. Study Population

This is a cross-sectional observational study in a tertiary university hospital in Hong Kong. From 21 August 2017 to 28 June 2019, 499 adult (age ≥18 year-old) Han Chinese subjects with LVH, defined as a maximal interventricular septal (IVST) and/or posterior wall thickness (PWT) ≥13 mm on echocardiography, were identified from our echo lab and prospectively recruited for FD screening in this study. Exclusion criteria were LVH caused by known sarcomere gene mutation, infiltrating cardiomyopathies (e.g., amyloidosis), athletic heart, non-compaction cardiomyopathy, and other non-FD metabolic or syndromic conditions associated with LVH. Conditions that cause afterload and/or preload increase such as hypertension and aortic/mitral valve regurgitation/stenosis were not exclusion criteria of this study. Informed consent was obtained from each patient and the study protocol conforms to the ethical guidelines of the 1975 Declaration of Helsinki as reflected in a priori approval by the institution’s human research committee. All authors have full access to all the data in the study and take responsibility for the integrity of the data and the accuracy of the data analysis.

### 2.2. Alpha-Galactosidase A Activity Assay and Genetic Study of the GLA Gene

A dried blood spot (DBS) assay of blood samples of all patients was performed to measure alpha-galactosidase A activity using the MS/MS method [13] as previously described by the biochemical lab in the National Taiwan University hospital [14]. Alpha-L-iduronidase (IDUA) was used as the control enzyme in duplication. For subjects with low plasma alpha-galactosidase A activity (defined as the IDUA/GLA ratio ≥ 10) [14], 3 mL of blood was collected and kept in an ethylenediaminetetraacetic acid tube for *GLA* gene sequencing using the Sanger method.

### 2.3. Demographic and Clinical Data Collection

Demographic data and patients’ medical history were reviewed from their medical charts. Han Chinese ethnicity subgroups (Teochew, Canton, Fujian, Shanghai, or others) were self-reported by the subjects. First-degree relatives of each genetically confirmed FD patient were invited to undergo familial screening.

### 2.4. Cardiac Evaluation

Standard 2D and Doppler transthoracic echocardiography was performed on all subjects. The following parameters were obtained based on the recommendation of the American Society of Echocardiography [15]: IVST and PWT at end-diastole, LV end-diastolic (LVEDD) and end-systolic dimensions (LVESD) were measured in the parasternal long-axis view; LV mass was calculated by the linear method and indexed to body surface area; LV end-diastolic (LVEDV) and end-systolic volumes (LVESV) were measured and LV ejection fraction (LVEF) was calculated using the biplane Simpson’s method. The LA volume (LAV) was obtained using the biplane area-length method at end-systole. The relative wall thickness (RWT) was calculated with the formula (2 × PWT)/LVEDD. All measurements were made in 3 cardiac cycles in sinus rhythm and 5 cardiac cycles in patients with atrial fibrillation. Endomyocardial biopsy was performed for all genetically confirmed FD patients who were going to start ERT subsidized by government funding in a protocolized way as stipulated by the healthcare policy in Hong Kong.

### 2.5. Statistical Analysis

Data are presented as mean ± SD, range, or number (percentage). Comparisons between patients with versus without genetically confirmed FD used Fisher’s exact test, chi-square test, or Student’s t-test where appropriate. Analyses were performed with SPSS 25.0 (IBM Corp., Armonk, NY, USA). A value of *p* < 0.05 was considered statistically significant.

## 3. Results

Table 1 summarizes the demographic, clinical, and echocardiographic characteristics of the study population. We studied 336 men and 163 women (mean age, 66.4 ± 12.7 years). The majority reported their ethnicity subgroup to be Canton (56.9%), followed by Teochew (17.0%) and Fujian (2.2%). Hypertension, diabetes and/or left-sided valve diseases were prevalent in 75.4%, 65.7%, and 22.4%, respectively, in the study population. The mean IVST was 15 ± 2 mm (range, 13−34 mm).

**Figure 1:**
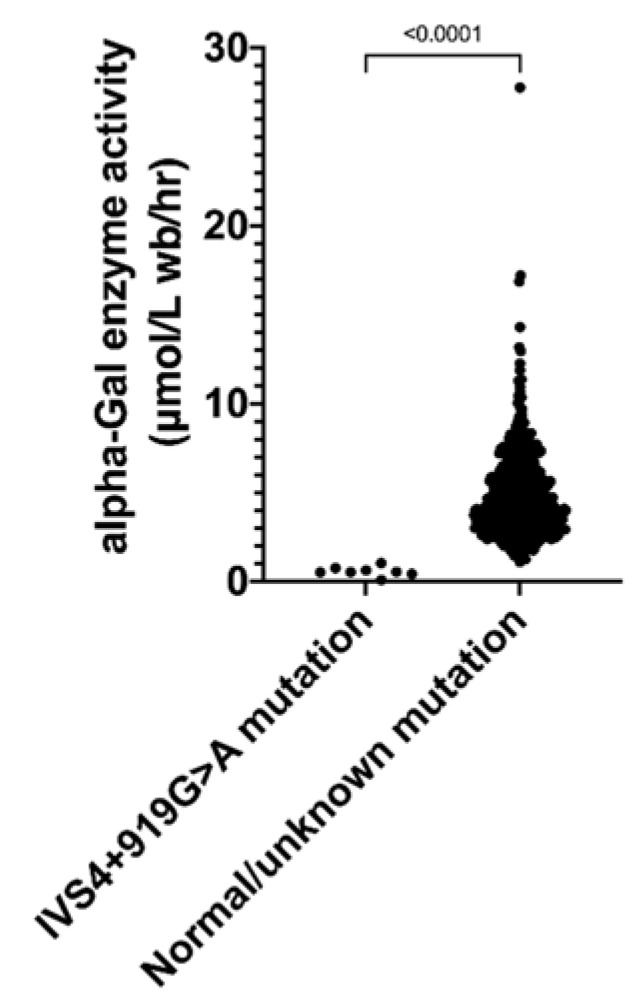
Scatter plot of alpha-Gal enzyme activity. Patients with IVS4 + 919G > A mutation had significantly lower plasma enzyme activity compared to those with normal/unknown mutation.

A low plasma alpha-galactosidase A enzyme activity (0.57 ± 0.27 μmol/L wb/h, *p* < 0.0001 vs. control; Figure 1) was found in eight unrelated patients (all men; age, 53−74 years). In all eight patients, we detected IVS4 + 919G > A mutation in the GLA gene. The prevalence of FD was herein 1.6% in the overall study population, and 2.4% in men. Patients with a known IVS4 + 919G > A mutation had different demographic distribution in ethnicity subgroups than non-FD patients (*p* = 0.0172), with 5 (62.5%) patients being Teochew people, 2 (25.0%) being Cantonese, and 1 (12.5%) being Wenzhounese. Heart failure was more common among patients with known IVS4 + 919G > A mutation than those without (*p* = 0.011), whereas other comorbid conditions showed no between-group differences. There were no differences in IVST and PWT between patients with or without known IVS4 + 919G > A mutations. The mean RWT in both LVH groups were ≥0.42 with no inter-group differences, consistent with concentric hypertrophy. FD patients had a larger LVEDV (*p* = 0.028). Echocardiographic features previously described as typical of FD, including binary appearance of endocardial border, hypertrophy of papillary muscles, myocardial hypertrabeculation/crypts, and elongated anterior mitral leaflet, were not detected in our FD patients. 

**Figure 2:**
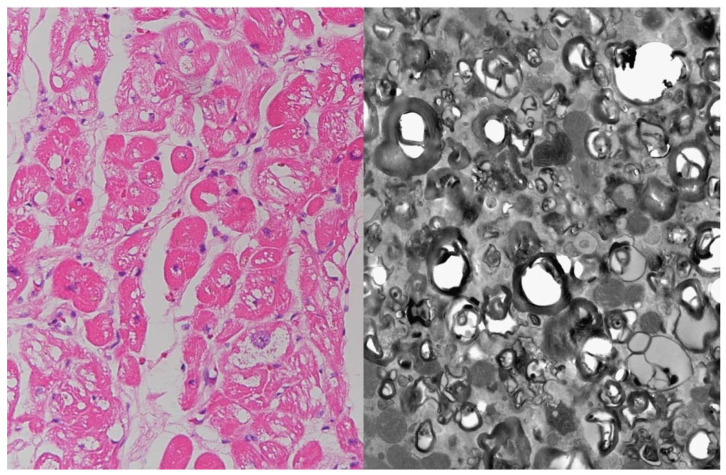
Endomyocardial biopsy specimen. Hematoxylin-eosin staining (×200) shows hypertrophic myocytes with vacuolization (**left panel**). Electron microscopy shows dense lamellar bodies consistent with cardiac involvement of FD (**right panel**).

Endomyocardial biopsy was performed in six patients with IVS4 + 919G > A mutation. Histologic sections revealed hypertrophic myocytes with the presence of vacuolization and electron microscopy showed dense lamellar bodies, consistent with cardiac involvement of FD (Figure 2). For the remaining two patients, endomyocardial biopsy was not performed; one patient refused the biopsy procedure; another patient was deemed ineligible for ERT due to clinically advanced cardiac disease and was treated conservatively. Cardiac involvement of FD in patients who did not undergo biopsy is only presumed as there are other possible causes of LVH.

Table 2 summarizes the clinical and demographic characteristics as well as the results of enzymatic and genetic study of each of the eight confirmed FD patients. None of the IVS4 + 919G > A mutation patients exhibited any classic neurological, ophthalmic, or dermatological FD symptoms. In contrast, they presented with heart failure or exertional dyspnea (*n* = 5), high-grade atrioventricular block requiring a pacemaker (*n* = 2), non-sustained ventricular arrhythmia (*n* = 1), chest pain (*n* = 3), and/or an incidental murmur (*n* = 1), with hypertension and/or severe valvular pathology evident in half of the patients. Kidney involvement was evident in two patients. The mean delay in the diagnosis of FD was 8.4 ± 4.9 years (range, 2−18 years).

**Figure 3:**
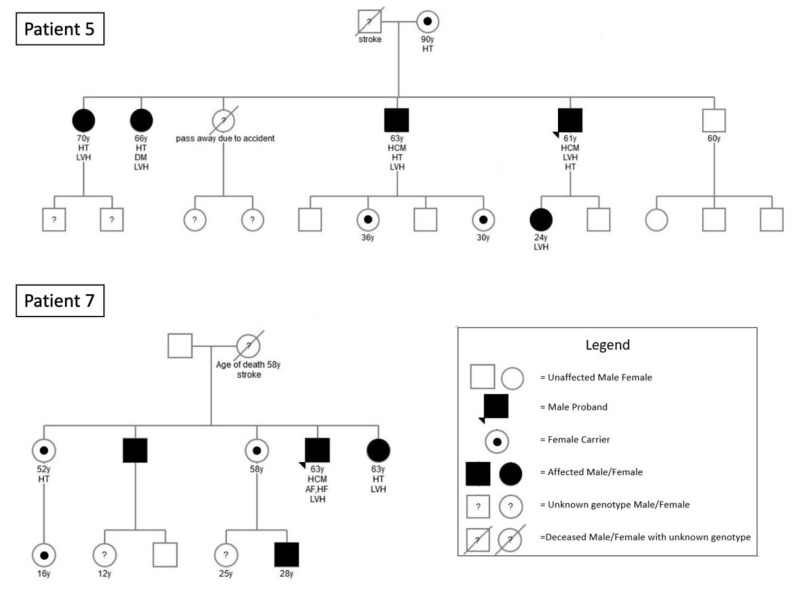
Pedigree of Index Fabry Patient 5 and 7. Circles are women and squares are men. Filled symbols are affected patients with GLA IVS4 + 919G > A mutation. Empty symbols are unaffected relatives without mutation. Dotted females are asymptomatic carriers. Dashed symbols are deceased family members. Question marks indicate family members with an unknown genotype as a genetic test was not performed. Numbers are ages. Index patients are indicated by triangles. AF = atrial fibrillation; DM = diabetes; HCM = hypertrophic cardiomyopathy; HF = heart failure; HT = hypertension; LVH = left ventricular hypertrophy.

All families were invited to participate in familial screening for FD. Figure 3 summarizes the results of the familial studies. Of the 2 families that agreed to undergo familial screening, a total of 12 additional family members were confirmed to have IVS4 + 919G > A mutation. Many family members were residing overseas or in mainland China and therefore did not participate in family screening in Hong Kong.

## 4. Discussion

The IVS4 + 919G > A mutation accounts for the largest portion of FD patients in the current global statistics [6,8,16,17,18]. Initially reported in Kyushu, Japan [19], this mutation was subsequently found to be highly prevalent on neonatal screening in Taiwan [8,16]. The present study is the first to systematically investigate the prevalence of FD among a non-selective Han Chinese patient population with LVH. Most previous FD screening studies in the LVH populations were predominantly targeted at non-Asian patients with unexplained and/or severe LVH [3,4,10,11,12,20,21,22,23,24,25,26,27,28,29]. Studies adopting a more all-inclusive screening approach generally yielded a low detection rate of less than 1% [30]. A small series of 16 Taiwan Chinese men who had been diagnosed with idiopathic hypertrophic cardiomyopathy found 4 cases with the IVS4 + 919G > A mutation [8]. In the present study, we found a relatively high prevalence of IVS4 + 919G > A mutation among a Chinese patient population of moderate LVH with many patients having co-existing hypertension and/or valve diseases. Despite including these patients, we detected an unexpectedly high prevalence of FD with the IVS4 + 919G > A mutation. In fact, the LVH in some of our patients have been attributed by their physicians to poorly controlled hypertension or severe aortic valve disease, leading to a delay in the FD diagnosis for years. Our findings suggest that FD screening among Chinese patients with LVH should not routinely exclude those with hypertension and valve disease.

IVS4 + 919G > A mutation of the *GLA* gene has been associated with late-onset cardiac variant of FD [16]. Of 620 adults with the IVS4 + 919G > A mutation identified through familial screening of 916,383 newborns in Taiwan, 67% of men and 32% of women older than 40 years had LVH [16]. Screening for FD among LVH patients may improve the outcome of these patients and their affected family members, as early diagnosis can lead to prompt treatment with ERT in eligible patients [18].

Given that classic symptoms including acroparesthesia, angiokeratoma, and hypohidrosis are typically absent in late-onset phenotypes [2], enquiry of these symptoms are not useful in screening FD for adult Chinese LVH patients. In our study, heart failure is more frequent in LVH patients with deficient alpha-Gal A enzyme activity and the IVS4 + 919G > A mutation than those with normal enzyme levels and no/unknown mutations. FD screening may be targeted at LVH patients with heart failure.

We found a disproportionally high prevalence of subethnic Teochew people among patients with the IVS4 + 919G > A mutation. The Teochew people are a Han Chinese people native to the historical Chaozhou prefecture (now the Chaoshan region) of the eastern Guangdong province who speak the Southern Min language (also the native language of most Taiwanese). Today, most Teochew people live in Hong Kong, Guangdong Province, Taiwan, and Southeast Asia. They can be found almost anywhere in the world, including North America, Australia and France [31]. A recent genetic study with haplotype analysis speculates that the hotspot Taiwanese IVS4 + 919G > A mutation originated from a founder traceable to Chinese ancestors over 800 years ago [9], and migration of decedents who inherited the mutation disseminated it to Central and Southern China, Taiwan, and possibly nearby countries including Japan. Future screening strategies for FD may target at the at-risk demographic patient populations including Chinese descents and the Teochew people.

**Figure 4:**
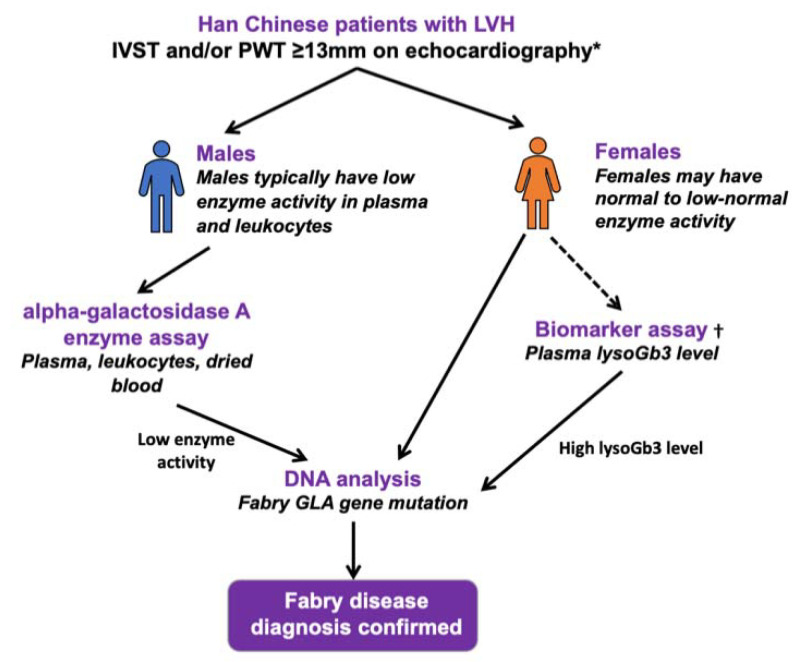
Proposed diagnostic flow chart for screening FD in men and in women with LVH. IVST = interventricular septal thickness; PWT = posterior wall thickness. * Potential red flags that should raise the clinical suspicion and prompt screening of FD in LVH patients include: Teochew people, heart failure, and arrhythmias; the presence of hypertension and/or aortic/mitral valve pathology should not exclude patients from FD screening. †Plasma lysoGb3 is a potential primary screening biomarker for FD in females.

Our study has limitations. First, DBS testing for alpha-galactosidase A activity may be insensitive in the female heterozygote [4]. This may have led to underestimation of the prevalence of FD among female patients in our patient population. Measurement of alpha-galactosidase A activity in heterozygous females is unreliable because heterozygotes (only one of the two x-chromosomes is affected) have variable levels of alpha-galactosidase A activity that can overlap with levels found in healthy controls. Thus, genetic testing is required to make the diagnosis of FD among females. A proposed diagnostic pathway for men and for women with suspected FD is shown in Figure 4. For logistic reason and budget concerns, our study protocol did not use genetic tests or a plasma lyso-Gb3 assay for screening female subjects. Nevertheless, the high prevalence of FD among male patients in a patient population of everyday practice revealed by DBS testing in our study is alarming. Secondly, only two families agreed to undergo familial screening, and not all family members in each family accepted the screening. Nevertheless, 12 additional family members were found to have the IVS4 + 919G > A mutation, allowing earlier evaluation in the younger affected family members.

## 5. Conclusions

There is a high prevalence of FD with *GLA* IVS4 + 919G > A mutation among Han Chinese patients with LVH in Hong Kong. Dried blood spot testing of enzyme levels is an effective screening method for FD in this patient population. While the true prevalence of FD among Han females is likely to be underestimated by enzyme screening, the high frequency of FD among Han males is confirmed in this study. Commonly acquired causes of LVH such as hypertension and valve disease, as they often co-exist with IVS4 + 919G > A mutation, should not exclude susceptible patients from being screened for FD in adult LVH patients, especially among Han Chinese.

## Figures and Tables

**Table 1:** Characteristics of the overall study population and comparisons between confirmed FD and non-FD patients.

Variables	Overall	FD	Non-FD	*p*
	(*n* = 499)	(*n* = 8)	(*n* = 491)	
**Demographic**				
Age, years	66.4 ± 12.7	63.0 ± 7.0	66.5 ± 12.8	0.211
Men, *n* (%)	336 (67.3)	8 (100)	328 (66.8)	0.058
Ethnicity subgroup, *n* (%)				0.0172
Canton	284 (56.9)	2 (25.0)	282 (57.4)	
Teochew	85 (17.0)	5 (62.5)	80 (16.3)	
Fujian	11 (2.2)	0 (0.0)	11 (2.2)	
Shanghai	5 (1.0)	0 (0.0)	5 (1.0)	
Others	27 (5.4)	1 (12.5)	26 (5.3)	
Unknown	8 (17.4)	0 (0.0)	87 (17.7)	
Diabetes, *n* (%)	328 (65.7)	1 (12.5)	170 (34.6)	0.274
Hypertension, *n* (%)	376 (75.4)	4 (50.0)	372 (75.8)	0.107
Aortic valve disease, *n* (%)	48 (9.6)	1 (12.5)	47 (9.6)	0.557
Mitral valve disease, *n* (%)	64 (12.8)	2 (25.0)	62 (12.6)	0.274
Heart failure,%)	63 (12.6)	4 (50.0)	59 (12.0)	0.011
Arial fibrillation, *n* (%)	112 (22.4)	4 (50.0)	108 (22.0)	0.080
Short PR interval (<120 ms), *n* (%)	8 (1.6)	0 (0.0)	8 (1.6)	1.000
IVST, mm	15 ± 2	18 ± 6	15 ± 2	0.118
PWT, mm	12 ± 3	13 ± 3	12 ± 3	0.255
LVEDD, mm	43 ± 7	49 ± 10	43 ± 7	0.177
LVESD, mm	30 ± 7	34 ± 9	29 ± 7	0.966
LVM, g	222 ± 73	355 ± 202	220 ± 67	0.100
LVMI, g/m^2^	129 ± 38	181 ± 94	128 ± 36	0.159
LVEDV, mL	93 ± 41	126 ± 34	92 ± 41	0.028
LVESV, mL	42 ± 21	62 ± 30	41 ± 27	0.098
LVEF, %	57 ± 10	53 ± 14	57 ± 10	0.371
LAV, ml	66 ± 39	78 ± 32	66 ± 40	0.326
RWT	0.57 ± 0.19	0.57 ± 0.19	0.57 ± 0.19	0.956

IVST = interventricular septal thickness; LAV = left atrial volume; LVEDD = left ventricular end-diastolic diameter; LVESD = left ventricular end-systolic diameter; LVEDV = left ventricular end-diastolic volume; LVESV = left ventricular end-systolic volume; LVEF = left ventricular ejection fraction; LVM = left ventricular mass; LVMI, left ventricular mass index; PWT = posterior wall thickness; RWT = relative wall thickness.

**Table 2:** Characteristics of patients with confirmed FD.

Patient	1	2	3	4	5	6	7	8
Age of diagnosis of FD, years	74	59	53	69	59	69	59	63
Gender	Male	Male	Male	Male	Male	Male	Male	Male
Ethnicity subgroup	Teochew	Canton	Wenzhou	Canton	Teochew	Teochew	Teochew	Teochew
Initial presentation	Poorly controlled hypertension since age 65; incidental murmur with severe aortic regurgitation at age 71	Exertional dyspnea and chest pain at age 49; hypertension; acute pulmonary edema with severe mitral regurgitation associated with chordal rupture at age 59, requiring mitral valve surgery	Dizziness with lacunar infarct/ischemic changes on MRI brain at age 46; diabetes; Exertional dyspnea at age 53	Hypertension and diabetes at age 51; syncope with third degree AVB requiring pacemaker at age 57; onset of AF and heart failure at age 67; NSVT requiring upgrade of pacemaker to ICD at age 69; echo showed septal hypertrophy IVDT = 16mm with posterior wall akinesia	Chest pain, non-ST MINOCA at age 53; hypertension; obstructive sleep apnea; labelled HCM	Heart failure with reduced ejection fraction and AF at age 65; treated as dilated cardiomyopathy possibly caused by alcoholism	Ischemic heart disease requiring percutaneous coronary intervention at age 48; obstructive sleep apnea; AF onset at age 59 requiring catheter ablation	age 61; chest pain and exertional dyspnea at age 62; labelled HCM
ECG findings	LVH with strain RBBB	LVH with strain	RBBB	AF, 3° AVB	LVH with strain	AF, LVH	AF, LVH	RBBB, LPFB, 2:1 AVB
IVST, mm	19	29	14	14	17	13	24	15
PWT, mm	13	17	12	13	19	10	10	13
LVEDD, mm	52	60	36	65	39	50	41	46
LVESD, mm	33	43	23	50	29	38	26	32
LVM, g	378	812	160	420	302	218	294	257
LVMI, g/m^2^	222	369	84	247	144	104	147	124
LVEF, %	60	40	73	31	45	49	65	59
GLA activity, μmol/L wb/h	0.55	0.43	0.09	0.54	0.76	0.64	0.52	1.04
IDUA/GLA ratio	27	11	18	27	10	10	10	10
Creatinine, µmol/L	204	116	89	118	87	74	98	83
eGFR, mL/min/1.73 m²	26	58	84	53	83	88	72	86
Proteinuria	Urine TP/Cr: 6.62 mg/mg Cr	Urine TP/Cr: 0.05 mg/mg Cr	Urine TP/Cr: 0.04 mg/mg Cr	Spot urine albumin: <0.3 mg/L	Urine TP/Cr: 0.05 mg/mg Cr	24 h urine protein: 0.13 g/24 h	Urine TP/Cr: 0.04 mg/mg Cr	24 h urine protein: <0.04 g/24 h
GLA gene mutation	IVS4 + 919G > A	IVS4 + 919G > A	IVS4 + 919G > A	IVS4 + 919G > A	IVS4 + 919G > A	IVS4 + 919G > A	IVS4 + 919G > A	IVS4 + 919G > A
ERT	Decided not for ERT due to clinically advanced kidney disease; patient refused renal and endomyocardial biopsy	Started	Started	Decided not for ERT due to clinically advanced cardiac disease	Started	Started	Started	Planned to start

AF = atrial fibrillation; AVB = atrioventricular block; ECG = electrocardiogram; eGFR = estimated glomerular filtration rate based on CKD-EPI equation; ERT = enzyme replacement therapy; HCM, hypertrophic cardiomyopathy; ICD = implantable cardioverter-defibrillator; IDUA= Alpha-L-iduronidase; IVST = interventicular septal thickness; LPFB = left posterior fascicular block; LVEF = left ventricular ejection fraction; LVEDD = left ventricular end-diastolic diameter; LVESD = left ventricular end-systolic diameter; LVH = left ventricular hypertrophy; LVM = left ventricular mass; LVMI, left ventricular mass indexed to body surface area; MINOCA = myocardial infarction with non-obstructive coronary artery; NSVT = non-sustained ventricular tachycardia; PWT = posterior wall thickness; RBBB = right bundle branch block; urine TP/Cr = urine total protein/creatinine ratio.

## Data Availability

Data are available upon request from the authors.
